# Sir William Hamilton's Lectures on Metaphysics
**Lectures on Metaphysics and Logic*. By Sir William Hamilton, Bart. Edited by the Rev. R. L. Mansel, B.D., and John Veitch, M.A. Vols. I. and II. Metaphysics. William Blackwood and Sons. 1859.


**Published:** 1859-07-01

**Authors:** 


THE JOURNAL

OF

PSYCHOLOGICAL MEDICINE

AND

MENTAL PATHOLOGY.

JULY 1, 1859.

### Art. I.—SIR WILLIAM HAMILTON'S LECTURES ON METAPHYSICS.*

From the vagueness and uncertainty of the meaning attached to the term *metaphysics* by various writers, and especially in popular estimation, it has been too frequently held as synonymous with all that is incomprehensible and unpractical; as something, the ultimate result of which could but be to bewilder and mislead. In some of the recognised acceptations of the term, perhaps this view may not be altogether unfounded. "With the Germans, metaphysics is a science purely speculative, which soars beyond the bounds of experience. The objects of this science are supersensual ideas, unattainable by experience; and the difficulty of defining the word lies in the circumstance that the very knowledge of the ideas sought requires some proficiency in the study. Hence to one altogether unacquainted with speculative philosophy, it is almost impossible to explain the meaning of the word 'metaphysics' as used in this sense. The very possibility of a science beyond experience has been denied by a great number of philosophers; and many works called metaphysical should rather be termed inquiries into the possibility of metaphysics."† With such definitions, such objects and aims of a so-called science, we can see that the celebrated *mot* of one of our Gallic neighbours was no less true than witty:—*Quand celui qui écoute n'entend rien, et celui qui parle n'entend plus, c'est metaphysique.*

But metaphysics in our English acceptation is a very different matter, and is synonymous with Philosophy proper—the science of Mind, in its phenomena and its laws. In this sense it becomes a real, practical, comprehensible, and important science;—inas-

* *Lectures on Metaphysics and Logic.* By Sir William Hamilton, Bart. Edited by the Rev. R. L. Mansel, B.D., and John Veitch, M.A. Vols. I. and II. Metaphysics. William Blackwood and Sons. 1859.

† *Penny Cyclopædia*, art. "Metaphysics."

much as all science is only such under conditions dependent upon the laws of mind; for "however great, and infinite, and various may be the universe and its contents,—these are known to us, not as they exist, but as our mind is capable of knowing them; *quicquid recipitur, recipitur ad modum recipientis*" (vol. i. p. 61).* In this aspect, we propose to follow Sir William Hamilton in his investigations, commencing as he does with the advantages of Philosophy—always bearing in mind that this is used synonymously with metaphysics, in the sequel.

The advantages of the cultivation of Philosophy are to be considered in two aspects, absolute and relative—absolute, in so far as it is immediately conducive to the mental improvement of the cultivator—relative, in proportion as its study is necessary for the prosecution of other branches of knowledge. Although our author confines his remarks altogether to the former, or absolute benefits of philosophy; and declines to enter "for the present" upon the question of how "its study is of importance to the Lawyer, the Physician, and above all to the Theologian;"† we must add a few words upon this, as it will explain fully why we consider it of importance to analyse with care this extensive work on so abstruse a subject as metaphysics. Mind has its laws—as certain, if not yet as definable as those of matter. Under certain conditions, inherent and external, it acts so as to produce certain results. The elements of calculation are more numerous and complex than those which appertain to physical laws—so complex as to produce results so varied, that the possibility of law is lost sight of, or questioned, in many minds. The law, however, exists; and the conditions being the same, the results will uniformly follow. It is manifestly, then, of the greatest importance to us to recognise and investigate these laws, in order that we may duly understand those departures from them which constitute the pathological phenomena of mind. It has often appeared to us, that it is owing to the want of this systematic comparison, that so many hitherto insoluble difficulties have been found in the way of the recognition of the more slight and obscure morbid phenomena connected with mental aberrations. When we have no fixed standard of comparison, departure from it is not easy to be defined. But if the laws of association, of memory, of will, &c. &c., be ever normally ascertained, and their conditions fully investigated, as we believe it may yet be possible to accomplish, we shall only require to bring to this test any mental phenomena that may be in question; and their accordance with, or departure

* The quotations, otherwise unacknowledged except by page marks, are always from the work under notice.

† Sir William never fully exhausted his own divisions of Philosophy; whilst some branches are treated at great length, others, amongst which is Ontology, or Metaphysics proper, are only very incidentally discussed.

from, clearly defined laws will be signalised,—not as readily as the perturbations of planetary motion, because of the greater complexity of the elements—but as truly, in proportion as we may have been able to form a perfect and unexceptionable standard. Thus we may test the efficiency of an organ of sense, by comparing it with a received standard; most persons see that a cherry is red, and the leaves of the tree green. If we present a cherry and a leaf to one who recognises no difference between them, save that of form, we know that the organ of sight, or its brain connexion, is in an abnormal condition. The chirp of a cricket is inaudible to some ears, which in other respects seem perfect; and at the other end of the scale some very deep musical notes are too grave to be heard by some ears, which exercise all their other functions perfectly. Knowing that the law is that these sounds are audible to the normal ear, we conclude that these ears are abnormal. The conditions here are simple, and easy of investigation; when we have to decide upon compound mental phenomena the difficulties become great. Whether a sufficiently defined standard of mental action can ever be attained, which will enable us to say with certainty that any mind not thus acting (within certain limits) is abnormal, we cannot decide. One thing is certain, that such a desirable consummation (and most desirable it is) can only be arrived at by an earnest search after, and accumulation of, whatever mental laws can be developed from any source; and the study of metaphysics is the one most essentially directed towards this end. Such are a few of the relative advantages of Philosophy, regarded as in connexion with our special ends—distant as yet, and difficult; but *not proved to be unattainable.*

The absolute advantages of the philosophy of mind are further subdivided by our author into the Subjective and the Objective utility;—the first, as it cultivates the mind, or "knowing subject," by calling its faculties into exercise;—the second, as it furnishes the mind with a certain complement of truths or objects of knowledge. The principal illustration of the *subjective* value of mental cultivation is derived from this consideration, that "*man is an end unto himself;*" and that the utility of a science is not to be calculated solely, or even chiefly, by the amount of money or bread which it will enable a man to earn; in other words, not by the extent to which it will make man an instrument, a means to another end; but by the extent to which it will make him individually perfect and happy. "It is manifest, indeed, that man, in so far as he is a mean for the glory of God, must be an end unto himself; for it is only in the accomplishment of his own perfection, that as a creature he can manifest the glory of his Creator" (p. 5). The training which recognises man as an end to himself and as a mean to some other end is different; the one is called Liberal, the other Professional education. The sciences concerned in the latter are called by the Germans *Brodwissenschaften*, or "Bread and Butter Sciences" (p. 6). "Even admitting then that the study of mind is of no immediate advantage in preparing the student for many of the subordinate parts in the mechanism of society; its utility cannot, on that account, be called in question, unless it be asserted, that 'man liveth by bread alone,' and has no higher destination than that of the calling by which he earns his subsistence." Human perfection and human happiness must coincide; and this perfection can only be accomplished by means of mental cultivation; and such studies as contribute to the perfection of the individual man as an end, and not as a mean to other ends, must be as truly considered "useful" studies, as those which enable man to earn his daily bread.

The objective utility of Philosophy depends upon manifold considerations. A knowledge of the human mind is "confessedly the highest and most interesting of all studies;" it is Philosophy *par excellence*. "On earth (says Phavorinus) there is nothing great but man; in man, there is nothing great but mind." Chilon asks of the oracle what is of all things the best?—"To know thyself" is the response. "The proper study of mankind is man," says our great Pope.

"But though mind, considered in itself, be the noblest object of speculation which the created universe presents to the curiosity of man, it is under a certain relation that I would now attempt to illustrate its dignity when viewed as the object through which, and through which alone, our unassisted reason can ascend to the knowledge of a God. The Deity is not an object of immediate contemplation; as existing, and in himself, he is beyond our reach; we can know him only mediately through his works, and are only warranted in assuming his existence as a certain kind of cause necessary to account for a certain state of things, of whose reality our faculties are supposed to inform us. The affirmation of a God being thus a regressive inference, from the existence of a special class of effects to the existence of a special character of cause, it is evident that the whole argument hinges on the fact,—Does a state of things really exist such as is only possible through the agency of a Divine Cause? For if it can be shown that such a state of things does not really exist, then our inference to the kind of cause requisite to account for it is necessarily null" (p. 25—6).

The author then proceeds fully to demonstrate that Theology is wholly dependent upon Psychology; because upon this depends the proof of the moral nature of man; and with this stands or falls the proof of the existence of a Deity. The argument concerning the superiority of the study of metaphysics over that of physics is summed up in the striking language of Jacobi:—

"*Nature conceals God;* for through her whole domain Nature reveals only fate, only an indissoluble chain of mere efficient causes without beginning and without end, excluding, with equal necessity, both providence and chance. An independent agency, a free original commencement within her sphere and proceeding from her powers, is absolutely impossible. Working without will, she takes counsel neither of the good nor the beautiful; creating nothing, she casts up from her dark abyss only eternal transformations of herself, unconsciously and without an end; furthering with the same ceaseless industry decline and increase, death and life—never producing what alone is of God, and what supposes liberty, the virtuous, the immortal.

"*Man reveals God;* for Man by his intelligence rises above nature, and in virtue of this intelligence is conscious of himself as a power not only independent of, but opposed to, nature, and capable of resisting, conquering, and controlling her. As Man has a living faith in this power, superior to nature, which dwells in him; so he has a belief in God, a feeling, an experience of his existence. As he does not believe in the power, so does he not believe in God; he sees, he experiences nought in existence but nature,—necessity,—fate" (*Von den Göttlichen Dingen*).

*What is philosophy?* Literally, a love of wisdom—a term apparently originated by Pythagoras, who, when asked by Leon what art he had chiefly studied, replied that he professed no art, and was simply a *philosopher*. He further stated, that whilst some men are in pursuit of honours, and others of riches, there are a few who, indifferent to all else, devote themselves to an inquiry into the nature of things. These are the students of wisdom, or philosophers. Socrates was probably the first to bring the name into common use. The definitions that have been given of philosophy are very numerous. The science of things human and divine, and of the causes in which they are contained; the science of effects by their causes; the science of sufficient reasons; the science of things possible, inasmuch as they are possible (Wolf); the science of things, evidently deduced from first principles (Descartes); the science of truths, sensible and abstract (Condillac); the application of reason to its legitimate objects; the science of the relation of all knowledge to the necessary ends of human reason (Kant); the science of the original form of the ego, or mental self (Krug), &c. &c., for an enumeration of which we refer to Lect. III., p. 50.

To define philosophy in general more clearly, our author divides knowledge into two kinds, empirical or historical, and philosophical. The former tells us that such and such things are, or have been; the latter tells us *how* and *why* they are. Civil history is an example of the former, natural history of the latter. Historical knowledge is the γνῶσις ὅτι ἔστι; philosophical knowledge is the γνῶσις διότι ἔστι, *cur res sit*, and may be termed knowledge of the cause, scientific, or rational knowledge. Philosophy is not content with a phenomenon, but inquires its *cause*. This cause, then, in turn becomes a phenomenon, and a source of discontent, until *its* cause is investigated. Philosophy thereby comes to be recognised as a search after first causes; and necessarily tends, not towards a plurality of ultimate or first causes, but towards one alone. This—the Creator—it can never reach as an object of immediate knowledge;

"But as the convergence towards unity in the ascending series is manifest, in so far as that series is within our view, and as it is even impossible for the mind to suppose the convergence not continuous and complete, it follows—unless all analogy be rejected,—unless our intelligence be declared a lie—that we must, philosophically, believe in that ultimate or primary unity which, in our present existence, we are not destined in itself to apprehend" (p. 60).

All the sciences are branches of philosophy; but, as has been before observed, because "man is the measure of the universe," and the mind is man, the science of the human mind is philosophy proper, philosophy *par excellence*. And thus philosophy in general is equivalent to a knowledge of things by their causes; whilst in its stricter and more defined meaning, it is confined to the sciences which constitute, or hold immediately of, the science of mind.

Lecture IV. treats of the *causes* of philosophy, of which we must be content with a brief enumeration. They are of two classes—essential, as contained in man's very capacity for knowledge; complementary and assistant, as resulting from certain feelings with which he is endowed. The first class comprises the innate tendency to search after causes; and as a necessary corollary to this, the search after unity. This latter is the guiding principle in philosophy, and all systems bear more or less the traces of it. This love of unity is a source of error, as in too hasty and extensive generalisations. The second class includes chiefly Wonder, which, combined with certain intellectual tendencies, becomes Curiosity, and is the chief accessory incentive to Philosophy.

The Fifth Lecture treats upon the dispositions with which philosophy ought to be studied. The first important point noticed is the renunciation of prejudice, from early teaching, from social errors, and from the influence of custom; and the influence of man on man in times of tranquillity and of convulsion is sketched. The author considers that men are the offspring of the times—not the times of the men. Had not the public mind been ripe for the changes, the fate of Luther and Zwingli in the sixteenth century would have been the same as that of Huss and Jerome of Prague, in the fifteenth.

"Woe to the revolutionist who is not himself a creature of the revolution! If he anticipate, he is lost; for it requires, what no individual can supply, a long and powerful counter-sympathy in a nation to untwine the ties of custom which bind a people to the established and the old" (p. 88).

These passages on the force of opinion and example are most interesting and suggestive; but we must not dwell upon them. The second practical condition of the pursuit of philosophy is the subjugation of the passions, especially sloth and pride. Bacon observes that "the eye of human intellect is not dry, but receives a suffusion from the will and from the affections, so that it may be almost said to engender any science it pleases. For what a man wishes to be true, that he prefers believing."

The *method* of philosophy is next discussed, and it is shown that there is and can be but one true method, that of *analysis*, including *synthesis;* for these are clearly demonstrated to be one, and indivisible. The one necessary condition of philosophy, or its possibility, is the decomposition of effects into their constituent causes; every effect being nothing more than the sum or complement of all the partial causes. A neutral salt, for example, is an effect compounded of three proximate causes—viz., an acid, an alkali, and the force which brought these two into the requisite approximation. The decomposition into causes is analysis; but "analysis without a subsequent synthesis is incomplete; it is a mean cut off from its end. Synthesis without a previous analysis is baseless; for synthesis receives from analysis the elements which it recomposes. Each is the relative and correlative of the other." Thus the two constitute only a single method, and the only possible one, of philosophy. Induction is shown to be synthetic in character; for the general principle includes many more facts than those analyzed, from which it was derived. All induction postulates the uniformity of nature's laws. The author concludes that the purity and equilibrium of these two elements (synthesis and analysis) constitute the perfection of philosophy, and that its aberrations have been all so many violations of the laws of this one method.

In Lecture VII. we find it stated that "the whole of philosophy is the answer to three questions: 1st. What are the facts or phenomena to be observed? 2nd. What are the laws which regulate these facts, or under which these phenomena appear? 3rd. What are the real results, not immediately manifested, which these facts or phenomena warrant us in drawing?"

With regard to the mind, the answer to the first question gives us the phænomenology of mind, empirical psychology, or the inductive philosophy of mind. These phenomena relate to three orders of mental development. (1.) Those of our cognitive faculties, or faculties of knowledge; (2) those of our feelings, or of pleasure and pain; and (3) those of our conative powers, or the phenomena of will and desire.

In like manner the answer to the second question gives us the Nomology of mind, under the same three divisions;—that of the cognitive faculties being represented by logic;—that of the feelings by what is called the æsthetic;—whilst the nomology of the conative powers involves Moral and Political Philosophy.

The solution of the third question gives us Ontology, or Metaphysics Proper—otherwise called Inferential Psychology;—it includes the *à priori* arguments for the Being of God, the Immortality of the Soul, &c.

The following is a tabular view of the distribution of Philosophy as here proposed:—

| | | |
|---|---|---|
| MIND or Consciousness affords | FACTS — Phænomenology, Empirical Psychology. | Cognitions.<br>Feelings.<br>Conative Powers (Will and Desire). |
| | LAWS—Nomology, Rational Psychology . . | Cognitions—Logic.<br>Feelings—Æsthetic.<br>Conative Powers—Moral Philosophy, Political Philosophy. |
| | RESULTS — Ontology, Inferential Psychology | Being of God.<br>Immortality of the Soul, &c. |

It is with the first of these departments, the Phænomenology of Mind, or Empirical Psychology, that we are at present solely concerned.

*Definition.*—Psychology is the science conversant about the phenomena or modifications, or states, of the mind, or *Conscious-Subject*, or *Soul*, or *Spirit*, or *Self*, or *Ego* (p. 129).

The *conscious-subject* is the mind, the individual, that which knows,—as distinguished from all else that can be known as an object of contemplation. But

"Mind and matter, as known or knowable, are only two different series of phenomena or qualities; mind and matter, as unknown and unknowable, are the two substances in which these two different series of phenomena or qualities are supposed to inhere. The existence of an unknown substance is only an inference we are compelled to make, from the existence of known phenomena; and the distinction of two substances is only inferred from the seeming incompatibility of the two series of phenomena to coinhere in one."

Our knowledge, therefore, whether of mind or matter, is all relative and phenomenal; of existence absolute, and in itself positive, we know nothing;—all that we know objectively is a collection of properties affecting our own consciousness; this latter is undefinable and insusceptible of analysis.

The number of the properties of existent things is not *of necessity* the same as the number of our powers of apprehension. Beyond these, we know and can assert the reality of no existence; but we are not warranted in denying such existence.

"The universe may be conceived as a polygon of a thousand sides, or facets; and each one of these may be conceived as representing one special mode of existence. Now of these thousand sides or modes, all may be equally essential; but three or four only may be turned towards us, or *be analogous to our organs*. One side or facet of the universe, as holding a relation to the organ of sight, is the mode of luminous or visible existence; another, as proportional to the organ of hearing, is the mode of sonorous or audible existence, and so on. But if every eye to see, if every ear to hear, were annihilated, the modes of existence to which these organs now stand in relation,—that which could be seen, and that which could be heard, would still remain; and if the intelligences reduced to the three senses of touch, smell, and taste, were then to assert the impossibility of any modes of being except those to which these three senses were analogous, the proceeding would not be more unwarrantable than if we now ventured to deny the possible reality of other modes of material existence than those to the perception of which our five senses are accommodated."

The inhabitant of Saturn is represented in Voltaire's "Micromegas" as recognising 300 essential properties of matter, having 72 senses, and living 15,000 years; and yet complaining bitterly of the pitiful boundaries of time, space, and perception with which he is hedged in. Micromegas himself, from the Dog-Star, has very nearly 1000 senses, with life 700 times longer than the other, and the elementary properties of matter proportionately more numerous; yet is no nearer to personal or philosophical contentment.

But however multiplied might be our powers or faculties, still our knowledge of mind and matter would be merely relative—a recognition of phenomena only: of existence itself we could still know nothing.

Another limit to our knowledge is this—that the very properties of existence are not known to us in their native purity, but are modified by our organs of perception and other circumstances. All our knowledge is a sum made up of several elements, and the great business of philosophy is to analyse and discriminate these elements, and to determine whence these contributions have been derived. Thus in seeing an object, we see it through a medium, in the first place, atmospheric or otherwise; and next through our organs of vision. How much of the object actually pictured to the mind then depends upon the real object itself,—how much upon the external medium,—and how much upon the organ of sense, forms a very abstruse question. Certainly nothing can be much more different, than the vibrations of light through the various tissues of the eye and the picture which they collectively form in the mind; or the motions of the various parts of the auditory apparatus, and the concert of sweet sounds into which the mind interprets them.

*Definitions of terms.*—SUBJECT is used to denote the unknown basis underlying the various phenomena or properties of which external or internal sense makes us aware; and in this sense is synonymous with *substance* in its philosophical acceptation. But in the modern philosophy *subject* is generally used to signify the basis of the various mental phenomena or operations. *Substance* is "a term for the substratum we are obliged to think to all that we variously denominate a *mode*, a *state*, a *quality*, an *attribute*, a *property*, an *accident*, a *phenomenon*, an *appearance*, &c." (p. 150). The two latter terms are used referring to a thing, *as known;* all the former are employed in reference to a substance, *as existing*. *Mode* is the manner of the existence of a thing, as a piece of wax may be round or square, or solid or fluid,—none of these being essential; *modes*, therefore, are variable states. *State* is nearly synonymous with *mode*, "but of a meaning more extensive, as not exclusively limited to the mutable and contingent." (p. 150).

*Qualities* are essential, and accidental. The essential are "those aptitudes, those manners of existence and action, which it cannot lose without ceasing to be;" as for instance in man, sense and intelligence; in body, dimensions; in God, eternity, omniscience, omnipotence, &c. The accidental are those which bodies may have at one time and not at another, as "the whiteness of a wall, the fineness of the weather;" or those which they always have, but might lose without ceasing to be; as the periodic movement of the planets. *Attribute* is properly convertible with *quality*, but is conventionally limited to qualities of a higher application,—as we speak of the qualities of matter, but of the attributes of intelligence.

*Property* is generally convertible with quality; *accident* is "an abbreviation for accidental or contingent quality."

*Phenomenon* is "that which appears," and is thus properly synonymous with *appearance*, but is used to express the same thing in more strict and philosophical sense.

We must give our author's account of MIND *in extenso*.

"In regard to the etymology of this term, it is obscure and doubtful; perhaps, indeed, none of the attempts to trace it to its origin are successful. It seems to hold an analogy with the Latin *mens*, and both are probably derived from the same common root. This root, which is lost in the European languages of Scytho-Indian origin, is probably preserved in the Sanscrit *mena*, to *know* or *understand*. The Greek *νοῦς*, *intelligence*, is, in like manner, derived from a verb of precisely the same meaning (*νοέω*). The word *mind* is of a more limited signification than the term *soul*. In Greek philosophy, the term ψυχὴ, *soul*, comprehends, besides the sensitive and rational principle in man, the principle of organic life, both in the animal and vegetable kingdoms; and in Christian theology it is likewise used, in contrast to πνεῦμα, or *spirit*, in a vaguer and more extensive signification.

"Since Descartes limited psychology to the domain of consciousness, the term mind has been rigidly employed for the self-knowing principle alone. Mind therefore, is to be understood as the subject of the various internal phenomena of which we are conscious, or that *subject* of which consciousness is the general phenomenon. Consciousness is, in fact, to the mind, what extension is to matter or body. Though both are phenomena, yet both are essential qualities; for we can neither conceive mind without consciousness, nor body without extension. Mind can only be defined *a posteriori*,—that is, only from its manifestations. What it is in itself, that is, apart from its manifestations, we, philosophically, know nothing; and accordingly, what we mean by mind is simply that which *perceives*, *thinks*, *feels*, *wills*, *desires*, &c." (p. 156, Lect. IX.).

*Conscious-subject.*—The act of consciousness is of the most elementary character, and evades description; but that is not required, as it is the one essential condition of all knowledge. But this consciousness is only a phenomenon, and presupposes a subject in which it inheres—a something that is conscious;—this is the *conscious-subject*,—"a brief, but comprehensive definition of mind itself."

*Object* is that about which the conscious-subject is conversant —that which is known—the *materia circa quam*, as *subject* is the *materia in quâ*. And as *subjective* is that which proceeds from, or belongs to, the thinking subject, so *objective* is that which proceeds from, or belongs to, the object known. The *subject* is the I, the Ego, the mind; the object is everything else, including body, organs, actions, and manifestations. For the mind contains the man, not the man the mind. "Thou art the soul," says Hierocles, "but the body is thine." And Cicero—"Mens cujusque is est quisque, non ea figura quæ digito demonstrari potest." The thoughts also are objective in so far as they are objects of consciousness and reflection, though subjective in origin—this is *subjective objectivity*.

*Hypothesis* is a provisional judgment of the mind, by which phenomena not as yet explicable are referred to some cause or class to which we imagine they may possibly belong, until we can permanently classify and prove their position; in obedience to the longing of the mind after unity. Hypothesis is only allowable on two conditions; the first involving the actual existence of the phenomena to be accounted for; the second, the impossibility of accounting for them except by an hypothesis, An hypothesis is good in proportion as it involves nothing contradictory or discordant with known facts—as it explains satisfactorily the facts—and as it is independent of subsidiary hypotheses. *Theory* is a vague term, indicating a practical evolution intellectually of an hypothesis, but opposed actually to practice, by being merely intellectual and not active.

We must pass over without notice the definitions of *Power, Faculty, Capacity, Disposition, Habit, Act, Operation, Energy, Functions,* &c.; their philosophical acceptation does not differ materially from the ordinary and conventional one.

Proceeding to the actual distribution of the mental phenomena, we find that consciousness is their one essential element; but that they are divisible into three grand classes, Knowing, Feeling, and Willing. Thus—

"I see a picture;—first of all, I am conscious of perceiving a certain complement of colours and figures—I recognise what the object is. This is the phenomenon of cognition or knowledge. But this is not the only phenomenon of which I may be here conscious. I may experience certain affections in the contemplation of this object. If the picture be a masterpiece, the gratification will be unalloyed; but if it be an unequal production, I shall be conscious, perhaps, of enjoyment, but of enjoyment alloyed with dissatisfaction. This is the phenomenon of feeling—or of Pleasure and Pain. But these two phenomena do not yet exhaust all of which I may be conscious on the occasion. I may desire to see the picture long,—to see it often,—to make it my own, and perhaps I may will, resolve, or determine to do so. This is the complex phenomenon of Will and Desire" (p. 184).

Will, desire, and aversion, presuppose knowledge and feeling, therefore the logical order of the mental phenomena is—first, Knowledge; second, Feeling; and third, Will and Desire (Conation).

Consciousness, as has been observed, is the one necessary condition of all these. It cannot be defined, yet the act in the aggregate admits of philosophical analysis, and contains as its elements—1st, A recognising or knowing subject; 2nd, A recognised and known modification; and 3rd, A recognition or knowledge by the subject of the modification. Consciousness and knowledge therefore mutually involve each other;—logically, that is; for it will become afterwards a matter for discussion whether in actuality they are always co-extensive—*i.e.* whether consciousness be always present with knowledge, and knowledge with consciousness.

Consciousness may be said to be "the recognition by the thinking subject of its own acts or affections." On this all are agreed; but as to its special conditions some are generally admitted, and some are subjects of controversy. Of those generally admitted, these are the chief;—actual knowledge—*immediate* (as distinguished from *mediate*) knowledge—discrimination—judgment—

and memory. For the development of these, we must refer the reader to Lecture XI., as we must hasten on with our analysis.

"The first contested position which I am to maintain is, that our consciousness is coextensive with our knowledge. But this assertion, that we have no knowledge of which we are not conscious, is tantamount to the other, that consciousness is coextensive with our cognitive faculties,—and that this again is convertible with the assertion, that consciousness is not a special faculty, but that our special faculties of knowledge are only modifications of consciousness. The question, therefore, may thus be stated—Is consciousness the genus under which our several faculties of knowledge are contained as species,—or, is consciousness itself a special faculty co-ordinate with, and not comprehending, these?" (p. 207).

Sir William answers the former question in the affirmative, and exposes at some length the errors of former writers on this subject. He also propounds as a fundamental axiom, that there can be no consciousness of a cognitive act, without a consciousness of its object; and that "it is palpably impossible that we can be conscious of an act without being conscious of the object to which that act is relative" (p. 212). He shows that imagination is a direct consciousness of certain ideas in the mind; and memory is also a direct consciousness of a condition of mind remaining from past impressions of events: it is not, as Reid represented, "an immediate knowledge of the past," but in philosophical propriety it is not a knowledge of the past at all, but a knowledge of the present, and a belief in the past.

We are here only concerned to give an exposition of our author's views, and not a critique; we may, however, remark that many of these positions admit of much dispute; and some are not altogether congruous with the subsequently evolved ideas.

Sir William proceeds to discuss Reid's views as to the perception of external objects. All philosophers, he says, before Reid, allowed to the mind no immediate knowledge of the external world. They conceded to it only a representative or mediate knowledge of external things, derived from the modifications produced by them in its own state. Reid's boldest stroke in philosophy was to assert that the mind had a direct and immediate recognition of external things themselves; but he then appeared

"to have been startled by his own boldness, and instead of carrying his principle fairly to its issue, by according to consciousness, on his doctrine, that knowledge of the external world as existing, which, in the doctrine of the philosophers, it obtained of the external world as represented; he inconsistently stopped short, split immediate knowledge into two parts, and bestowed the knowledge of material qualities on perception alone, allowing that of mental modifications to remain exclusively with consciousness. Be this, however, as it may, the exemption of the objects of perception from the sphere of consciousness can be easily shown to be self-contradictory" (p. 224).

The author then proceeds to argue that we are directly conscious of external objects, and that the contrary view involves a general absurdity; because—

"An act of perception is an act of knowledge; what we perceive, that we know. Now, if in perception there be an external reality known, but of which external reality we are, on Reid's hypothesis, not conscious, then there is an object known of which we are not conscious; but as we know only inasmuch as we know that we know,—in other words, inasmuch as we are conscious that we know,—we cannot know an object without being conscious of that object as known; consequently, we cannot perceive an object without being conscious of that object as perceived."

This is a statement both in matter and manner much too important to be passed over without comment. In this part of the course Sir William strongly asserts the doctrine of our immediate consciousness of the external world. Now, if this means anything, it means that the evidence we have of external things is the same in kind as that which we have of our own minds; plainly, as it appears to us, inconsistent with the previous views promulged concerning the influence of media and organs upon the objects of knowledge. The image of a tree painted upon the retina, after the rays of light have been many times refracted in the atmosphere, and in passing through the various humours of the eye, affords the same *kind* of evidence to the mind of its existence, as an emotion or a desire in the mind itself. Surely this cannot be; if so, we must be said on the same general principles to be directly conscious of Saturn's ring, and the satellites of Herschel. Certainly we may *arbitrarily call* this kind of knowledge *consciousness;* but in so doing we must make consciousness include the results of all manner of perception and investigation, and ignore the special meaning of the term altogether—a proceeding which would obscure all metaphysical reasoning to the very uttermost, and render exactitude of terminology an unattainable desideratum. A second objection that we have to this view is derived from an after part of the course, where the author broadly asserts that the evidences of consciousness, and the phenomena revealed to it, are essentially and without appeal true. If we receive these two dicta, we as pathologists shall be compelled to recognise the reality of spectral illusions, the truth of dreams, and the veritable existence of all manner of fanciful phenomena; for all these are essentially manifestations of consciousness; but of this more anon.

We have alluded to the manner of this statement. In it Reid is falsely and sophistically represented. Reid never asserted that we perceived a thing of which we were not conscious; but only that perception was *the* faculty in operation, with regard to the external world; accompanied, as he elsewhere states all the mental faculties, especially perception, to be, by consciousness; without which the whole argument would be too futile for even a child to indulge in. We dwell particularly upon this, because we think that almost the only fault of these excellent lectures is the tendency to prove that Reid was wrong in everything: that Stewart and Brown have mistaken and misrepresented him; and that he has mistaken and misrepresented everybody else.

Sir William so plainly states our *consciousness* of external objects, that he speaks of being "conscious of the ink-stand" (p. 228), as a more proper phrase than "being conscious of the perception of the ink-stand." He admits the strangeness of the sound, but avers that very slight consideration will show its correctness.

Reflection and attention are then shown on the same principles to be general phenomena of consciousness. The argument is too elaborate and involved for abstraction. We refer to Lect. XIII. One point incidentally introduced in it we must notice in passing, viz., that Sir William upholds, against the opinion of Stewart and others, that the mind is capable of attending to more than one object at once. Stewart, and the philosophers of his school, hold that the attention can only be directed to one object at one time, and explain all the phenomena that appear to prove the reverse, by the theory of the rapid transition of thought. Even in listening to harmonies, this theory is maintained. Thus Stewart writes:—

"It is commonly understood, I believe, that in a concert of music, a good ear can attend to the parts of the music separately, or can attend to them all at once, and feel the full effect of the harmony. If the doctrine, however, which I have endeavoured to establish be admitted, it will follow that in the latter case the mind is constantly varying its attention from one part of the music to the other, and that its operations are so rapid as to give us no perception of the intervals of time."

Stewart holds the same theory with regard to sight; and that everything that is seen or heard is only seen or heard by a rapid succession of the *minimum visibile* and *minimum audibile* through the mind. This appears in contradiction to all reason, and indeed appears to be *à priori* theory run mad. Sir William Hamilton very properly controverts this, and his reasoning is forcible. As to music, he says:—

"This example appears to amount to a reduction of his opinion to the impossible. What are the facts in this example? In a musical concert we have a multitude of different instruments and voices emitting at once an infinity of different sounds. These all reach the ear at the same indivisible moment in which they perish, and consequently, if heard at all, much more if their mutual relation or harmony be perceived, they must be all heard simultaneously. This is evident. For if the mind can attend to each minimum of sound only successively, it consequently requires a minimum of time in which it is exclusively occupied with each minimum of sound. Now, in this minimum of time, there coexist with it, and with it perish, many minima, which, *ex hypothesi*, are not perceived, are not heard, as not attended to. In a concert, therefore, on this doctrine, a small number of sounds only could be perceived, and above this petty maximum, all sounds would be to the ear as zero. But what is the fact? No concert, however numerous its instruments, has yet been found to have reached, far less to have surpassed, the capacity of mind and its organ" (p. 243).

The phenomena of sight are similarly investigated, but at too great length to admit of abstraction. Much of this reasoning and counter-reasoning might have been spared to metaphysicians, if they had but considered that psychology must be an empirical science, one of observation and experience; and that to attempt to set aside the plain testimony of all experience by abstract *à priori* argument upon the supposed incapacity of a simple element, like mind, to be in two states at one time, or any other incomprehensible formula, is not philosophy, but a darkening of counsel by words without knowledge. Our author considers it fully demonstrated that the mind can embrace more than one object at the same time, and inquires how many? By Charles Bonnet the mind is allowed to have a distinct notion of six objects at once; Abraham Tucker allows only four; Destutt Tracy allows six. Sir William agrees with this opinion, and gives some not very conclusive illustrations. A valuable analytic attention can only be given to one object at a time in ordinary cases; and the more diffused the attention, the less will be the practical result.

To proceed, Attention (*auct. loquent.*) is consciousness applied to an act of will or desire under a particular law. "This law, which we call the law of limitation, is, that the intension of our knowledge is in the inverse ratio of its extension; in other words, that the fewer objects we consider at once, the clearer and more distinct will be our knowledge of them." Attention is not always and essentially a voluntary act. We are frequently determined to an act of attention, as to many other acts, independently of our free and deliberate volition. Nor is attention always controllable; it cannot always be commanded, nor can it always be withdrawn. If we are occupied intently, a clock may strike, or we may be spoken to, without the attention being aroused; but we cannot *intentionally and with will* remain in this state of unconsciousness. We may close our eyes or shut our ears, but we cannot, with our organs unobstructed, wholly refuse attention at will. Attention is of three degrees or kinds. The first, a mere vital and *irresistible* act; the second, an act determined by desire, which, though voluntary, may be resisted by our will; the third, an act determined by a deliberate volition. This last is the most valuable, and in its highest degree stamps the mind of the greatest efficiency and power. It is difficult at the commencement, but admits of almost indefinite cultivation. Sir William quotes a number of high authorities to prove the pre-eminent excellence of the faculty of voluntary attention, and more than hints that genius is nothing more than a high development of the faculty. He remarks that the difference between an ordinary mind and the mind of a Newton, consists principally in this, that the one is capable of a more continuous attention than the other; that a Newton is able without fatigue to connect inference with inference in one long series towards a determinate end; while the man of inferior capacity is soon obliged to let fall the thread which he had begun to spin. Bacon also places all men of equal *attention* on one level, recognising nothing as due to genius. Helvetius goes so far as to say that genius is indeed nothing but a continued attention (*une attention suivie*). Buffon also speaks of it as a protracted patience. "In the exact sciences, at least (says Cuvier), it is the patience of a sound intellect, when invincible, which truly constitutes genius." Lord Chesterfield acknowledges that the power of applying an attention, steady and undissipated, to a single object, is the sure mark of a superior genius.

This faculty has been manifested more or less by all whose names are associated with the progress of the intellectual sciences, and often has a tendency to degenerate into a habit akin to disease. The most characteristic illustrations are found amongst names which have made the world's mental history. Archimedes was, at the taking of Syracuse, so absorbed in a geometrical problem, that he merely exclaimed to the soldier who was about to kill him, *Noli turbare circulos meos.* Newton's absence of mind is well known: he frequently forgot to dine, and it is said he on one occasion used a lady's finger as a tobacco-stopper. It is said that Joseph Scaliger was so engrossed in the study of Homer during the massacre of St. Bartholomew, that he was only aware of his own escape from it on the next day. Carneades had to be fed by his maid-servant, to prevent him from starving. Cardan was wont, on a journey, to forget both his way and his object, and could not be roused from his thought to answer any questions. Alcibiades relates of Socrates that he once stood a whole day and night, until the breaking of the second morning, with a fixed gaze, engrossed with the consideration of a weighty subject; "and thus (he continues) Socrates is ever wont to do when his mind is occupied with inquiries in which there are difficulties to be overcome. He then never interrupts his meditation, and forgets to eat and drink and sleep—everything, in short, until his inquiry has reached its termination, or, at least, until he has seen some light in it." The mathematician Vieta was sometimes so absorbed in meditation, "that he seemed for hours more like a dead person than a living, and was then wholly unconscious of everything going on around him." The great Budæus forgot his wedding-day, and was found deep in his Commentary, when sought up by the party.

The forgetfulness of time is a very common event during abstraction; of this the instance already given of Socrates is almost equalled by that of a modern astronomer (quoted by Dr. Moore), who passed the entire night observing some celestial phenomenon; and being accosted by some of his family in the morning, he said—"it must be thus; I will go to bed before it is late."

Perhaps the insensibility to pain is the most remarkable of all the phenomena connected with abstraction. Pinel relates of a priest that in a fit of mental absence, he was unconscious of the pain of burning; the same is stated of the Italian poet Marini. Cardan relates something analogous concerning himself.

Malebranche does not hesitate to call attention the "*force of intellect;*" but in these extreme developments it becomes a disease which is not unlikely ultimately to destroy the intellect entirely.

In Lecture XV., Sir William supports the dogma that the testimony of consciousness is the criterion of all knowledge, and that this criterion is unerring (p. 266); and, as we have before observed, the reception of this idea would lead us to strange conclusions. It will be remembered that the author considers that we are immediately conscious of the external world; he now further states (p. 283) that "the absolute and universal veracity of consciousness must be maintained." How does this accord with the phenomena of dreaming, of illusions and hallucinations of the senses? All these involve acts of consciousness; and indeed, at p. 269, it is stated that *every* mental phenomenon must be considered a fact of consciousness. It may be answered that these are fanciful; but this is no philosophical answer. Sir William says that if we doubt one datum of consciousness, we must doubt all; because we have no criterion of truth but consciousness, and we must not reject consciousness on the authority of consciousness (*vide* Lect. XV. *passim*). He certainly gives certain limiting laws "under which consciousness may be applied to the consideration of its own phenomena"

(p. 268); but we cannot see that they exclude the force of our objection. These laws are—1st. That no fact be assumed as a fact of consciousness but what is ultimate and simple—the law of Parcimony; 2nd. That the whole facts of consciousness be taken without reserve or hesitation, whether given as constituent or regulative data—the law of Integrity; 3rd. That nothing but the facts of consciousness be taken, or if inferences of reasoning be admitted, that these at least be recognised as legitimate only as deduced from, and in subordination to, the immediate data of consciousness, and every position rejected as illegitimate which is contradictory of these—the law of Harmony.

That there may be no room to doubt his meaning, Sir William clearly distinguishes between the testimony of consciousness as a *fact*, and as an *evidence*. "In the case of a common witness, we cannot doubt the fact of his personal reality, nor the fact of his testimony as emitted; but we can always doubt the truth of what his testimony avers" (p. 271). It is in this latter sense that he contends for the full and unquestionable credibility of consciousness. Unless we have grievously misunderstood his argument, this appears to us one of the most startling mistakes ever made in philosophy; and the most singular part of the matter is this—that these obvious objections are never once alluded to.

Lecture XVI. treats of the various hypotheses to account for the phenomenon of dual consciousness—*i. e.*, the consciousness of a Me and a Not Me—a *self*, and a something external to self; and analyses the different theories of the identity of, or distinction between, matter and mind. Perhaps to all these, the remark may be appropriate, that Sir William applies to a part only—viz., that "the mutual polemic of these systems is like the warfare of shadows; as the heroes in Valhalla, they hew each other in pieces, only in a twinkling to be reunited, and again to amuse themselves in other bloodless and indecisive contests."

All natural systems of philosophy, *i. e.*, all systems that are not transcendentally incomprehensible, recognise two distinct, and in some measure opposed, orders of existence—those of mind and matter, or body. But these have a constant intercourse, and perpetual mutual reactions. How is this accomplished? How can the immaterial act upon the material? How, above all, can matter affect that which is immaterial—spirit or mind? This is a profound difficulty, one against which the wings of speculation have been broken again and again—one hitherto unsolved, most probably insoluble, by human reason. It will not appear less obscure if we briefly pass in review a few of the most famous theories that have been invented, to give an appearance of explanation to this great mystery.

The first in order which we shall notice is called the "system of Assistance or Occasional Causes," belonging to Descartes, Malebranche, and the Cartesians generally. It sets out with setting forth the apparent impossibility of any actual communication between a spiritual and a material substance, and hypothecates the perpetual immediate interposition of the Divine assistance. As the world was originally created by his will, so it owes its continuance from moment to moment only to the unremitted perseverance of the same volition :—

"God is thus the necessary cause of every modification of body, and of every modification of mind, and his efficiency is sufficient to afford an explanation of the union and intercourse of extended and unextended substances. External objects determine certain movements in our bodily organs of sense, and these movements are by the nerves and animal spirits propagated to the brain. The brain does not act immediately and really upon the soul; the soul has no direct cognizance of any modification of the brain ; this is impossible. It is God himself, who, when movements are determined in the brain, produces analogous modifications in the conscious mind. The body is not, therefore, the real cause of the mental modifications ; nor the mind the real cause of the bodily movements. The organic changes, and the mental determinations, are nothing but simple conditions, and not real causes; in short they are occasions, or occasional causes."*

This hypothesis did not satisfy Leibnitz, and he proposed instead that of Pre-established Harmony. According to this view, in brief, the mental and the physical world may be compared to two pieces of Divine mechanism, each entirely independent of the other, but so adjusted and regulated that the emotions, desires, and cognitions of the one always correspond chronologically to certain appropriate actions of the other. So, when I will to move my arm, the will has no action upon the limb, but the action takes place, because its time had arrived in the mechanism, as the time for willing it had arrived in the mind; and when a misfortune produces grief apparently, the one has no real causative connexion with the other ; but the two occur in succession because it was so arranged in the two systems from the beginning. Probably this was never regarded as anything more, even by the author, than an example of ingenuity. Any serious refutation of it is equally needless and impracticable.

The third hypothesis is a very feeble one—that of a Plastic Medium between soul and body—something that is neither one nor the other, but can hold intercourse with both. This merely adds a third element of difficulty to the already sufficiently irreconcileable two.

* Laromiguiére, "Leçons de Philosophie," tom. ii. p. 255—6.

The fourth theory is that of Physical Influence, but is only a statement of facts, that external objects *do* affect our senses, and produce changes which the soul perceives and acts upon accordingly; there is no attempt at explanation of the *modus in quo* of the "mysterious union of an extended and an unextended substance." In the words of Pascal, "Man is to himself the mightiest prodigy of nature; for he is unable to conceive what is body, still less what is mind, but least of all is he able to conceive how a body can be united to a mind; yet this is his proper being." And when all is said, we have to conclude that *magna immo maxima pars sapientiæ est, quædam æquo animo nescire velle.*

In Lecture XVII. the important question is discussed, Is the mind always consciously active? Not, of course, Have we always a memory of our consciousness? for that would at once be decided in the negative; also, from the consideration, Sir William Hamilton excludes states of coma, &c., about which experiment will tell us nothing. The question refers chiefly to states of sleep and somnambulism, and is this, Is the mind, so far as we can make it matter of observation, always in a state of conscious activity? It has, for the most part, been discussed theoretically, in reference to the active nature of mind. Sir William attempts to prove it by analogical observation. Plato and Aristotle, and their schools, for the most part believed in the continual energy of intellect. Cicero says, *Nunquam animus cogitatione et motu vacuus esse potest*; and St. Augustin in like manner, *Ad quid menti preceptum est, ut se ipsam cognoscat, nisi ut semper vivat, et semper sit in actu.* Descartes made the essence, the very existence of the soul, to consist in actual thought. Locke seems to have been the first to oppose these views.

"I confess myself (says he) to have one of those dull souls that doth not perceive itself always to contemplate ideas; nor can conceive it any more necessary for the soul always to think than for the body always to move; the perception of ideas being (as I conceive) to the soul, what motion is to the body; not its essence, but one of its operations."

Locke's opinion is good and philosophical. His illustrations, however, are not so apt, nor so free from vulnerable points, as might be wished; for he seems to think it improbable "that the soul in a sleeping man should be this moment busy a-thinking, and the next moment, in a waking man, not remember, nor be able to recollect, one jot of all those thoughts;" yet, that such is a very frequent case, is most indisputable, witness many of the phenomena of sudden waking, of somnambulism, &c. Sir William inclines to the view that the mind is always active; and that when it appears not to have been so, it is owing to the break in the train of thought, and forgetfulness of the ideas. He supports the opinion very ably by arguing from the well-known phenomena of forgetting dreams, and from the double life in an habitual somnambulist, where all that has passed in one state is totally forgotten in the other, but remembered again on the resumption of that condition. By analogy, he concludes that this same oblivion accounts for all apparent cessations of thought. But surely this is a too hasty induction. It must remain, after all, a matter for observation; for to reason *à priori* from some supposed essential property of mind, is begging the whole question at issue. A person is asleep, and on waking is not conscious of having had any ideas during his sleep; it is not sufficient to tell him that he must have had ideas, *because* he and others have on previous occasions had them and forgotten them. We incline to the opinion of Locke, not only as most in accordance with common experience, but as appearing to us most philosophical; also for another reason stated in a note.*

The question discussed in the Eighteenth Lecture is one of a highly interesting and important character. It is, Whether the mind is ever unconsciously modified? *i. e.*, whether it exerts energies, and is the subject of modifications, of neither of which it is conscious. Sir William decides it in the affirmative; though we, fully agreeing with him, can scarcely see how he reconciles this with his previous elaborate proof in Lect. XII., that our "consciousness is always co-extensive with our knowledge." In the investigation three degrees of mental latency are recognised. First: we know a science or language, not merely at the time that we make a temporary use of it, but inasmuch as we can apply it when or how we will; and thus the knowledge is at times latent.

"The second degree of latency exists when the mind contains certain systems of knowledge, or certain habits of action, which it is wholly unconscious of possessing in its ordinary state, but which are revealed to consciousness in certain extraordinary exaltations of its powers. The evidence on this point shows that the mind frequently contains whole systems of knowledge, which, though in our normal state they have faded into absolute oblivion, may, in certain abnormal states, as madness, febrile delirium, somnambulism, catalepsy, &c., flash out into luminous consciousness, and even throw into the shade of unconsciousness those other systems by which they had, for a long period, been eclipsed and even extinguished" (p. 340).

* The writer has a very distinct remembrance of his *first dream,* and that when he was at an age in some measure to reason upon it. So striking was the effect of the phenomenon upon his mind, and so astonished was he at the new world of thought opened to him, that it would be extremely difficult to convince him that the same had been occurring to him in all sleep ever since he was born, only that he had forgotten it. Why forget all the former, and remember this one so vividly? for the dream itself was of the most trivial and unconnected character.

The cases adduced in illustration of this, are those well-known instances, with which practical psychologists are so familiar, of languages being spoken in delirium and allied states, which in the healthy state the patient knew nothing of; but which, on investigation, have proved to have been heard at an early period of life, and apparently totally forgotten. Perhaps the most remarkable is that related by Coleridge in his *Biographia Literaria.* It is of a young woman, who, being seized with a nervous fever, in its course talked incessantly in Latin, Greek, and Hebrew. With great difficulty, tracing back her past life, it was discovered that many years before, when a child, she had lived with an old Protestant pastor, who was in the habit of walking up and down his kitchen, reading aloud out of his favourite authors. Of course, until this was known, the phenomenon was accounted a diabolic one, as she was what the people called a heretic.

The third degree of latency involves the whole pith and disputed matter of the question. Are there, in ordinary, mental modifications, *i. e.*, mental activities and passivities, of which we are unconscious, but which manifest their existence by effects of which we are conscious? Sir William not only answers this affirmatively, but does "not hesitate to maintain, that what we are conscious of is constructed out of what we are not conscious of—that our whole knowledge, in fact, is made up of the unknown and the uncognisable." (p. 348). This is curiously and ingeniously illustrated at great length; first, from the phenomena of perception. In sight we recognise a *minimum visibile*, which is the smallest expanse that can be seen, *i.e.*, which can consciously affect us.

"This being understood, it is plain that if we divide this *minimum visibile* into two parts, neither half can, by itself, be an object of vision, or visual consciousness. They are, severally and apart, to consciousness as zero. But it is evident that each half must, by itself, have produced in us a certain modification, real though unperceived; for as the perceived whole is nothing but the union of the unperceived halves, so the perception,—the perceived affection itself of which we are conscious,—is only the sum of two modifications, each of which severally eludes our consciousness" (p. 350).

The same mode of reasoning applied to the other senses seems to prove that all the knowledge which we acquire through our external senses, is made up of an infinity of incognisables or unknowables.

The illustration from the law of Association of Ideas is also striking. A, B, and C, are three thoughts—A and C do not immediately suggest each other, but each is associated with B, so that A suggests B, and B suggests C. But it may happen that thought C immediately follows A; how is this anomaly to be explained? By the principle of latent modification;—A suggests C, not immediately, but through B;—but as B, like the half of the minimum visibile, or minimum audibile, does not rise into consciousness, so we are apt to consider it as non-existent. Again, if a number of billiard balls be placed in a row, touching each other, and if a ball be made to strike in the line of the row, the ball at one end of the series, the motion is transmitted through the intermediate balls, to the one at the opposite end of the series, and this alone is impelled onwards, all the rest remaining where they were. Sir William gives one of his own personal experiences on this matter of association.

"Thinking of Ben Lomond, this thought was immediately followed by the thought of the Prussian system of education. Now, conceivable connexion between these two ideas in themselves, there was none. A little reflection, however, explained the anomaly. On my last visit to the mountain, I had met upon its summit a German gentleman, and though I had no consciousness of the intermediate and unawakened links between Ben Lomond and the Prussian schools, they were undoubtedly these,—the German—Germany,—Prussia,—and these media being admitted the connexion between the extremes was manifested" (p. 353).

Another class of unconscious modifications are the operations resulting from our acquired Dexterities and Habits.

"To explain these, three theories have been advanced. The first regards them as merely mechanical or automatic, and thus denying to the mind all active or voluntary intervention, consequently removes them beyond the sphere of consciousness. The second again, allows to each several motion a separate act of conscious volition; while the third, which I would maintain, holds a medium between these, constitutes the mind the agent, accords to it a conscious volition over the series, but denies to it a consciousness and deliberate volition in regard to each separate movement in the series which it determines" (p. 356).

To explain all these phenomena Stewart hypothecates consciousness without memory, a theory which Sir William controverts with great ingenuity and philosophical acumen, principally on the ground that consciousness and memory are always in the direct ratio of each other. This is true, but still not quite accordant with the author's views as expressed a few lectures before on the forgetting of dreams, &c. But viewed as a stern philosophical unity, these lectures must be acknowledged to present many of these inconsistencies.

From these general phenomena of consciousness follow as corollaries three facts—Self-Existence,—Mental Unity,—and Mental Identity;—points upon which we cannot at present enlarge. The Nineteenth Lecture concludes with a few remarks on the difficulties and facilities of psychological investigation, which we will briefly enumerate. The difficulties arise from 1st. the conscious mind being at once the observing subject, and the object observed; 2nd. from the want of mutual co-operation, inasmuch as mental analysis requires solitude rather than society; 3rd. from the fact that no fact of consciousness can be taken at second hand, but must be personally observed; 4th. that the phenomena of consciousness can only be studied through memory, as they cannot be arrested during observation; 5th. that the phenomena of the mental world are presented only in succession, and not side by side, as those of the external world; 6th. that they naturally blend with each other, and are presented in complexity; 7th and lastly, that the acts of reflection are not accompanied with the frequent and varied sentiment of pleasure, which we experience from the impression of external things. The facilities of philosophical study are also peculiar, and depend upon the simplicity of the requirements for the pursuit, the phenomena and all the means of investigation being always within reach.

This completes the analysis of the *general* phenomena of consciousness, contained in the first volume. The next treats upon the special manifestations of consciousness, in Perception, Memory, Association, Imagination, Judgment, Reasoning, &c. The consideration of these must be deferred to another opportunity.

---

